# Dendritic Cells in Oncolytic Virus-Based Anti-Cancer Therapy

**DOI:** 10.3390/v7122953

**Published:** 2015-12-09

**Authors:** Youra Kim, Derek R. Clements, Andra M. Sterea, Hyun Woo Jang, Shashi A. Gujar, Patrick W. K. Lee

**Affiliations:** 1 Departments of Pathology, Dalhousie University, Halifax, NS B3H 1X5, Canada; youra.kim@dal.ca (Y.K.); derek.clements@dal.ca (D.R.C.); 2 Department of Biology, Dalhousie University, Halifax, NS B3H 1X5, Canada; andra.sterea@dal.ca; 3 Department of Microbiology and Immunology, Dalhousie University, Halifax, NS B3H 1X5, Canada; hy405107@dal.ca; 4 Department of Strategy and Organizational Performance, IWK Health Centre, Halifax, NS B3K 6R8, Canada

**Keywords:** dendritic cells, tumor microenvironment, oncolytic virus, anti-viral immunity, anti-tumor immunity, immunotherapy

## Abstract

Dendritic cells (DCs) are specialized antigen-presenting cells that have a notable role in the initiation and regulation of innate and adaptive immune responses. In the context of cancer, appropriately activated DCs can induce anti-tumor immunity by activating innate immune cells and tumor-specific lymphocytes that target cancer cells. However, the tumor microenvironment (TME) imposes different mechanisms that facilitate the impairment of DC functions, such as inefficient antigen presentation or polarization into immunosuppressive DCs. These tumor-associated DCs thus fail to initiate tumor-specific immunity, and indirectly support tumor progression. Hence, there is increasing interest in identifying interventions that can overturn DC impairment within the TME. Many reports thus far have studied oncolytic viruses (OVs), viruses that preferentially target and kill cancer cells, for their capacity to enhance DC-mediated anti-tumor effects. Herein, we describe the general characteristics of DCs, focusing on their role in innate and adaptive immunity in the context of the TME. We also examine how DC-OV interaction affects DC recruitment, OV delivery, and anti-tumor immunity activation. Understanding these roles of DCs in the TME and OV infection is critical in devising strategies to further harness the anti-tumor effects of both DCs and OVs, ultimately enhancing the efficacy of OV-based oncotherapy.

## 1. Introduction

Currently available cancer treatment options such as surgery, chemotherapy, radiation, and hormone therapy, remain inadequate in consistently producing optimum outcomes from cancer [1]. Hence, a major focus of cancer research activities worldwide is concentrated on finding novel therapeutic modalities with enhanced anti-cancer benefits. Consequently, emerging evidence has identified the critical importance of the immune system in regard to cancer immune surveillance [2]. Research thus far shows that the presence of a functional anti-tumor T cell response directly correlates with positive cancer outcomes [3,4]. In this context, cancer immunotherapies, capable of promoting the generation of anti-tumor immunity, have garnered tremendous interest for the treatment of cancers of almost every origin. Interestingly, the establishment and regulation of such anti-tumor immune responses require the presentation of tumor antigens by dendritic cells (DCs) to tumor-specific T cells [5]. Considering the potential of DCs to influence both innate and adaptive immunity, therapeutic modulations involving DCs represent an ideal immunotherapeutic candidate to achieve clinically relevant beneficial immune responses against cancer.

DCs constitute a heterogeneous population of professional antigen-presenting cells (APCs), which can uptake, process, and present different types of antigens [6]. The inherent function of DCs to present tumor antigens is imperative in the generation of anti-tumor immunity as they can interact with various immune cells to initiate and maintain innate and adaptive immune responses [7]. However, several immunosuppressive mechanisms in the tumor microenvironment (TME) impair DC functions and block the development of anti-tumor immunity [8], which may decrease the efficacy of immunotherapies. Thus, efforts are underway to circumvent tumor-associated immunosuppression.

One of the most promising treatment options to overcome such obstacles is oncolytic virus-based anti-cancer therapy (oncotherapy). Oncolytic viruses (OVs) preferentially target and kill cancer cells through direct oncolysis and OV-induced anti-tumor immunity [9]. Immunosuppression in the TME provides an infection-vulnerable niche that is permissive for viral replication, causing direct lysis of the cancer cells. Following this OV-induced immunogenic cancer cell death with the resulting “danger” signals, there is an increased infiltration of APCs and lymphocytes in the TME and the consequent activation of non-specific and specific anti-tumor immunity with possible long-term protection against cancer recurrence [10]. In addition, OVs invoke other immunological events that override the impairment of tumor antigen presentation and promote the interaction between APCs and tumor-specific T cells [11]. Hence, better understanding DC-OV interaction is pivotal in devising novel or improved therapeutic approaches to harness the anti-tumor effects of both DCs and OVs, ultimately enhancing the efficacy of OV-based anti-cancer therapy. This review will summarize the role of DCs in anti-viral and anti-tumor immunity, and highlight how the latter can be initiated and sustained through cooperative DC-OV interactions.

## 2. Development and Function of DC Subsets

DCs originate from bone marrow hematopoietic progenitor cells, committed to either lymphoid or myeloid lineage, and reside in both peripheral and lymphoid tissues, where they are involved in immune surveillance and activation of T cell immune responses, respectively [12]. In mice, the heterogeneous population of DCs can be divided into two major groups: classical and non-classical (extensively reviewed by Mildner and Jung [13]). Classical DCs (cDCs) can be distinguished based on the expression of cell surface markers CD8, CD103, and CD11b. CD8+ cDCs reside in lymphoid tissues and are functionally specialized for cross-presentation [14]—the presentation of exogenous antigens by class I major histocompatibility complex (MHC) molecules [15]. Thus, CD8+ cDCs play a critical role in immune responses against viruses and intracellular bacteria. CD103+ cDCs, on the other hand, populate non-lymphoid tissues, particularly the intestinal mucosa where they regulate immune tolerance to commensal bacteria and food antigens [16]. Both CD8+ and CD103+ cDC lineage development is governed by the same transcription factors: inhibitor of DNA binding 2 (Id2) [17], interferon regulatory factor 8 (IRF8) [18], basic leucine zipper transcriptional factor ATF-like 3 (BATF3) [19], and nuclear factor, interleukin 3 regulated (NFIL3) [20]. In addition, CD11b+ cDCs make up the most abundant population of cDCs in lymphoid organs, and they can be further divided into CD4+ cDCs (CD8−, CD11b+, CD4+) and double negative cDCs (CD8−, CD11b+, CD4−) [21]. The development of CD11b+ cDCs is under the control of transcription factors Relb [22], NOTCH2 [23], RBP-J [24], IRF2 [25], and IRF4 [26]. CD11b+ cDCs possess prominent class II MHC presentation machinery and are thus important in the induction of CD4+ T cell responses [27].

Conversely, non-classical DCs consist of three major subsets: Langerhans cells, monocyte-derived DCs, and plasmacytoid DCs. Langerhans cells (LCs) are DCs in the epidermal skin layer with a prominent sentinel role, continuously sampling the tissue environment even in the absence of infection [28]. LCs migrate to lymph nodes via the afferent lymphatics and present antigens to activate naïve T cells. Monocyte-derived DCs (moDCs), also known as inflammatory DCs, originate from monocytes, which circulate in the blood and mature into tissue-resident macrophages; but under inflammatory conditions, they can also differentiate into DCs [29]. moDCs are similar to cDCs in terms of their expression patterns of class II MHC molecules, CD11b, and CD11c; however, moDCs also express CD64 which indicates their monocytic lineage [30]. Lastly, plasmacytoid dendritic cells (pDCs) in the blood and peripheral organs are key determinants of anti-viral innate immunity. Due to the constitutive expression of IRF7, pDCs can produce large amounts of type I interferons (IFNs) during viral infections and establish an anti-viral state [31,32]. They can also secrete pro-inflammatory chemokines and cytokines for the recruitment of leukocytes and regulation of immune responses. For instance, interleukin (IL)-12 secretion by pDCs can induce IFN-γ production in natural killer (NK) cells, CD4+ and CD8+ T cells [33,34,35], while IL-6 can promote the production of anti-viral antibodies by plasma B cells [36]. While pDCs have been shown to induce antigen-specific immune responses [37], they can also be poor stimulators of T cells owing to their low expression levels of MHC and co-stimulatory molecules [38]. In fact, it has been suggested that pDCs can induce CD4+CD25+ T regulatory (Treg) cells [39] or anergy in antigen-specific CD4+ T cells, where T cells remain unresponsive to antigens [40].

## 3. DCs in Viral Infections

### 3.1. DC Activation and Maturation

DCs are found in both “immature” and “mature” states, which differ in localization, phenotype, and function. Tissue-resident immature DCs efficiently capture antigens via several mechanisms. They engulf pathogens and cell debris by phagocytosis [41,42], and take up particles and extracellular fluid by micropinocytosis [43]. In addition, they employ receptor-mediated endocytosis via C-type lectin receptors [43] or Fc (Fragment, crystallisable region of antibodies) receptors [44] for antigen uptake. Immature DCs also express low levels of class I and II MHC molecules, as well as co-stimulatory molecules such as CD80 and CD86 [45,46,47]. However, upon pathogen recognition or stimulation by activatory signals (*i.e.*, pro-inflammatory cytokines [48]), DCs undergo a phenotypic change and migrate from peripheral to lymphoid tissues [49]. This migration is aided by the upregulation of C-C chemokine receptors (CCR), such as CCR2 and CCR7 which bind monocyte chemotactic protein 1 (MCP-1) and macrophage inflammatory protein-3-β (MIP-3β), respectively [50,51,52]. DCs complete their maturation in the secondary lymphoid organs, where the downregulation of phagocytic and endocytic receptors, upregulation of MHC and co-stimulatory molecules, and reorganization of the cytoskeleton make mature DCs more efficient at antigen presentation for T cell priming [47,53,54].

Activation of DCs can be induced directly by infectious agents and indirectly by inflammatory products. Pattern-recognition receptors (PRRs) such as Toll-like receptors (TLRs) on DCs recognize pathogen-associated molecular patterns (PAMPs)—microbial products conserved within groups of pathogens [55,56]—and induce a pro-inflammatory response that contributes towards the activation of DCs themselves as well as other innate immune cells [57]. For example, herpes simplex virus-2 (HSV-2) recognition by murine pDCs is mediated by TLR-9, triggering the secretion of IFN-α by pDCs [58]. Specifically, TLR-9 recognizes the unmethylated CpG motifs present on HSV-2 genomic DNA. Of note, DC subsets express different levels and types of TLRs, suggesting that they preferentially recognize distinct classes of pathogens [59]. While TLR-3 is absent in murine pDCs, it is preferentially expressed by CD8α+ DCs; similarly, TLR-7 is expressed by pDCs but not CD8α+ DCs [59]. In addition, DCs can detect viral infections via non-TLR cytosolic PRRs. For instance, cytosolic enzyme protein kinase R (PKR) can recognize viral double-stranded RNA (dsRNA) and induce type I IFN secretion by cDCs without TLR-3 involvement [60,61]. Another TLR-independent pathway for the recognition of intracellularly replicating viruses involves retinoic acid-inducible gene 1 (RIG-I), a dsRNA helicase enzyme that detects viral genome [62]. Furthermore, activatory signals can originate from other cells responding to PAMPs via their own PRRs [63]. For example, DCs are activated by IFNs secreted by virally infected cells, and LCs can be activated by tumor necrosis factor α (TNFα), IL-1, and IL-18 secreted by keratinocytes [64]. On the other hand, PAMP-independent activation of DCs depends on “danger” signals, also known as danger- or damage-associated molecular pattern (DAMPs), which are released by necrotic cells [65]. These host-derived endogenous molecules, such as heat shock proteins, hyaluron degradation products, ATP, and bradykinins, may mimic PAMPs and can thus trigger DC activation [66,67,68]. Once again, indirect activation is possible, wherein recognition of these stress molecules by healthy cells triggers the secretion of inflammatory mediators, such as TNFα, leading to DC activation.

### 3.2. Induction of Anti-Viral Immunity

DCs contribute towards anti-viral innate immune responses through activation of innate immune cells, such as NK cells, and cytokine production. Upon pathogen recognition in peripheral tissues, DCs produce chemokines, such as MCP-1, MIP-1α, and macrophage-derived chemokine (MDC) [69,70]. These signaling proteins recruit innate immune cells including macrophages, NK cells, NKT cells, and neutrophils to sites of infection, which then recognize and respond to pathogens through non-specific mechanisms. In particular, the interaction between DCs and NK cells is important in early defenses against infections through the production of other key effector molecules. For instance, DCs and NK cells have complementary functions, with DCs producing IL-18 or type I IFNs and presenting antigens, and NK cells producing IFN-γ and exhibiting cytotoxic functions [71,72,73]. In fact, CD11b+ DCs infected with murine cytomegalovirus (MCMV) were efficient at activating NK cells, while DC-derived IL-18 and IFN-α were necessary for NK cell cytotoxicity [74]. Similarly, IL-12 and IL-18 secreted by CD8α+ DCs were essential for the expansion of NK cells during acute MCMV infection while the presence of NK cells in return maintained the CD8α+ DC population in the spleen [75].

Moreover, as a major source of type I IFNs, DCs play a critical role in the establishment of an anti-viral state. By inducing an anti-viral state in cells, type I IFNs restrict viral replication and stimulate the production of anti-viral, anti-proliferative, and immunomodulatory proteins [76]. For example, CD8α+ DCs were identified as a potent producer of type I IFNs (IFN-α/β) during MCMV infections which limited viral replication; however, the same response was not observed after lymphocytic choriomeningitis virus (LCMV) infection, suggesting that DC-derived cytokines regulate immune responses in only certain viral infections [77]. Furthermore, as mentioned above, pDCs secrete type I IFNs, as well as IL-12 and IL-6, to regulate immune responses in NK cells, CD4+ and CD8+ T cells, and plasma B cells [33,34,35,36]. Thus, DCs activate and interact with the cells of the innate immune system, which in turn influence the nature of the adaptive immune response that follows.

One of the main roles of DCs in the induction of adaptive immunity is antigen presentation. The captured antigens are processed by the endocytic pathway of DCs and loaded onto MHC molecules [78,79]. MHC class II-rich compartments in immature DCs allow rapid presentation of exogenous antigens for the generation of CD4+ T helper cells [44,45]. Of note, different DC subsets induce different T helper (Th) cell polarization; CD8α+ DCs preferentially induce Th1 responses while CD8− DCs trigger the development of Th2 responses [80]. For the activation of cytotoxic CD8+ T cells, DCs present endogenous antigens via class I MHC molecules following direct infection as observed during infections by influenza viruses [81] and HSV [82]. For instance, DCs infected with influenza A virus expressed viral proteins HA and NS1 which induced potent cytotoxic T lymphocyte (CTL) responses [83]. This virus-DC interaction occurred with retention of cell viability and substantial production of IFN-α, and allowed for the processing of newly synthesized viral proteins by the traditional class I MHC pathway in the cytoplasm of DCs. For antigens from viruses that do not directly infect DCs, DCs can utilize the cross-presentation machinery, but the exact mechanism through which this occurs is still poorly understood [15,84].

The priming of anti-viral T cell responses requires three signals: (1) peptide-MHC complexes on DCs interacting with antigen-specific T cell receptors [7], (2) co-stimulatory molecules such as CD80, CD86 on DCs interacting with CD28 on T cells [53], and (3) inflammatory cytokines that stimulate and support T cell expansion and differentiation [85]. The nature of the PAMP-PRR interaction is an important determinant in the generation of signals 2 and 3 [80]. That is, the recognition of PAMPs by PRRs leads to increased expression of co-stimulatory molecules on DCs, contributing to T cell proliferation, differentiation, and survival. Without such co-stimulation, T cell anergy or immune tolerance may result instead of T cell activation. Furthermore, particular PAMPs will induce different types of signal 3, such as those resulting in the polarization of either a Th1 or Th2 response. Once generated, anti-viral CTL responses protect against subsequent infections. For instance, mice immunized with DCs pulsed with LCMV-specific peptide GP33-41 developed LCMV-specific anti-viral immunity which protected against LCMV challenge up to 60 days post immunization [86]. However, it is important to note that DCs contribute to not only the induction of anti-viral immunity but also the propagation of viral infections [87]. For example, DCs can serve as reservoirs for human immunodeficiency virus (HIV) and transport the virus to lymph nodes, contributing to the pathogenesis of the disease, while concurrently triggering anti-viral immunity [88]. Thus, the dual role of DCs in viral infections is a key factor to take into consideration when devising strategies to enhance or dampen anti-viral immune responses.

## 4. DCs in the Tumor Microenvironment

Clinical data from patients have conclusively established a positive correlation between anti-tumor CD8+ T cell immunity and patient survival, tumor grade, and disease outcome [89,90,91]. Anti-tumor immunity involves the recognition of class I MHC-peptide complexes on tumor cells by cytotoxic CD8+ T cells [92,93]. As such, many efforts are underway to identify tumor-associated antigens (TAAs), which are present on tumor cells as well as non-malignant cells, and tumor-specific antigens (TSAs), which are unique to individual tumors [94]. A critical step in the generation of anti-tumor immunity is the presentation of such tumor antigens by DCs resulting in the activation of tumor-specific T lymphocytes. In spontaneous priming of anti-tumor CD8+ T cells, DNA released from necrotic tumor cells can trigger the production of IFN-β by DCs through the STING pathway [95,96], wherein type I IFN signaling may contribute to innate immune responses against tumors [97]. For the establishment of anti-tumor immunity via therapeutic modulation, tumor-bearing mice immunized with DCs loaded with tumor antigens *ex vivo* have developed protection against tumor growth and reduction in the size of established tumors [98], and such DC-based cancer therapeutics have been used in clinical trials since the mid-1990s. As a case in point, MCA-207 sarcoma or MT-901 breast carcinoma cell lysate-pulsed DCs have been shown to prime CD8+ T cells, resulting in rejection of subsequent tumor challenge and reduction in pulmonary metastases [99]. Moreover, it has been demonstrated that CD8α+ DCs acquire tumor antigens *in vivo* by recognizing and binding exposed actin filaments of necrotic cells via the receptor DNGR-1 (CLEC9A) [100,101,102]. It is also possible to use DNA vaccines (*i.e.*, plasmid-based immunization) to deliver tumor antigens to DCs [103]. This therapeutic approach indirectly relies on DCs to present tumor antigens for the induction of anti-tumor immunity. In addition to mediating innate and adaptive anti-tumor immunity, DCs can have direct tumoricidal activity through perforin, granzyme B, and TNF-related apoptosis-inducing ligand (TRAIL) [104]; and promote innate anti-tumor immune responses through NK cell cytotoxic activity and IFN-γ production. Overall, these various functions of DCs are important in the generation of anti-tumor immune responses.

The generation of anti-tumor immunity is aided by the infiltration of DCs into the TME. Tumor cells produce chemokines, such as MIP-3α, which recruit immature DCs via CCR6 [105]; and after capturing tumor antigens, DCs migrate to secondary lymphoid tissues to initiate T cell responses against the tumor. Accordingly, increased levels of tumor-associated DCs (TADCs) are linked to favorable prognosis and prolonged survival of patients with various types of cancers including oral [106,107], lung [108,109], gastric [110], and colon [111]. It has also been shown that FMS-like tyrosine kinase 3 ligand (Flt3L)-mediated mobilization of DCs into murine fibrosarcoma led to decreased tumor growth and complete tumor regression [112]. Similar observations were made in patients with melanoma and renal cancer, where Flt3L administration resulted in increased numbers of DCs and monocytes, and allogeneic T-cell proliferation [113]. Therefore, the association between TADCs and anti-tumor immunity suggests the importance of increasing DC numbers and function in primary tumor lesions in order to promote positive cancer outcomes.

### Phenotypic and Functional Alterations of DCs

The immunosuppressive nature of the TME, however, is not conducive for the anti-tumorigenic properties of DCs. Tumor and tumor stromal cells, such as inflammatory infiltrate, fibroblasts, endothelial cells, and pericytes, create the TME which effectively promotes tumor progression and tumor immune evasion [114]. This is achieved through various cytokines, chemokines, growth factors, prostaglandins, and gangliosides. For instance, nitric oxide synthase 1 (NOS1) and arginase 1 (ARG1) expression by myeloid-derived suppressor cells (MDSCs) inhibit CD8+ T cell activation [115]; similarly, IL-10, transforming growth factor β (TGF-β), and cytotoxic T-lymphocyte-associated protein 4 (CTLA-4) expression by Tregs suppress the proliferation of T lymphocytes [116,117,118]. Moreover, tumor-associated macrophages (TAMs) promote angiogenesis [119], and TGF-β expression by cancer-associated fibroblasts induces epithelial-mesenchymal transition (EMT) [120], which is important in the initiation of metastasis [121]. Hence, the TME favors evasion of anti-tumor immunity and thus dampens the pro-tumorigenic properties of immune cells.

Consequently, the immunosuppressive local milieu influences the phenotype and function of TADCs, decreasing the allostimulatory capacity of these cells in generating anti-tumor immune responses (extensively reviewed by Gottfried, Kreutz, and Mackensen [122]). First, TADCs may display reduced capacity to capture, process, and present antigens [123,124]. For instance, DCs infiltrating renal cell carcinoma showed decreased antigen uptake, possibly due to tumor-derived vascular endothelial growth factor (VEGF) inhibiting phagocytosis [125,126]. Second, the TME inhibits the maturation of TADCs, which may consequently express less co-stimulatory molecules [127]. With an immature phenotype, such as the lack of CD80 and CD86 on DCs infiltrating colon cancers [127], TADCs may induce T cell tolerance, resulting in anergy and decreased proliferation of allogeneic T cells [128]. In addition, immature DCs can trigger deletional tolerance, resulting in apoptosis of antigen-specific T cells [129]. The differentiation of DCs can also be inhibited by macrophage colony-stimulating factor (M-CSF) and IL-6, as observed in renal cell carcinoma [130]. These tumor-derived factors favor the differentiation of hematopoietic progenitor cells towards a monocyte lineage that has reduced expression of MHC and co-stimulatory molecules. Given that MDSCs and DCs share a common hematopoietic origin, it has been suggested that the common progenitor may preferentially differentiate into MDSCs rather than DCs, thereby augmenting the immunosuppressive microenvironment [131]. Similarly, gangliosides, which are lipids shed by tumor cells following hypoxia-induced aberrant ganglioside composition, can also impair DC maturation, resulting in downregulation of antigen-processing machinery and co-stimulatory molecule expression [132]. Third, the migration of TADCs to secondary lymphoid tissues may be inhibited. This may be due to the immature state of TADCs [105] or directly mediated by tumor-derived TGF-β [133]. Ultimately, these tumor-induced dysfunctions of TADCs contribute to tumor immune evasion by inhibiting DCs from activating tumor-specific T cell responses.

Furthermore, TADCs can have tolerogenic functions through the expression of co-inhibitory molecules, programmed death-ligand 1 (PD-L1) and indoleamine-pyrrole 2,3-dioxygenase (IDO). For instance, PD-L1 is upregulated on ovarian cancer-infiltrating DCs, thereby suppressing tumor-specific T cells upon interacting with the programmed cell death protein 1 (PD-1) receptor [134]. The immunomodulatory enzyme IDO is expressed by pDCs isolated from melanoma patients, and its expression is upregulated by tumor-derived prostaglandin E2 (PGE2) [135]. IDO promotes CD8+ T cell anergy and CD4+ Treg differentiation [136], and it can also increase IL-10 production by TADCs [137,138]. TADCs are also capable of producing TGF-β [139] and display reduced levels of IL-12 production [140]. In addition, DCs can produce IL-23, which may suppress NK cell cytotoxic activities mediated by perforin and IFN-γ, thus promoting tumor development and metastasis [141]. Many such inhibitory activities of TADCs are driven by the transcription factor FOXO3; indeed, DCs isolated from prostate cancer patients display elevated levels of FOXO3 and effectively induce T cell tolerance [142]. Therefore, DCs in the TME can be polarized to an inhibitory state that contributes to tumor immune evasion.

In addition to tumor-induced dysfunction of DCs, there can also be elimination of functional DCs. Abnormal frequency of TADCs has been observed in melanoma patients with reduced number of LCs [143,144], and apoptosis has been suggested as the mechanism through which DCs are removed from the TME [145,146,147]. Similarly, other tumor-derived factors, such as gangliosides [148], mucin 2 (MUC2) [149], and high-mobility group protein B1 (HMGB1) [150], have been shown to trigger apoptosis of TADCs. This decrease in DC number appears to be a systemic effect as it is observed beyond the TME. For example, patients with breast, lung, pancreatic, liver, cervical, and head and neck cancers have a reduced number of DCs circulating in the blood, which is associated with poor prognosis [151,152,153,154,155]. Overall, TME-associated dysregulation of DC function and apoptosis of functional DCs is known to contribute towards the impaired induction of anti-tumor immunity.

## 5. Interaction between DCs and OVs

As indicated above, DCs are pivotal in the generation and sustainability of anti-viral and anti-tumor immune responses, and are recognized as the predominant contributors to bridging innate and adaptive immunity. To this end, numerous cancer immunotherapeutic approaches focus on the activation and accumulation of functional DCs or utilize DC-based vaccines (TAA/TSA-loaded DCs) to sufficiently mediate a tumor-specific T cell immune response. Although current cancer immunotherapies, either cancer vaccines or checkpoint blockade inhibitors, have had recent success in clinical practice, it would be ideal to have an immunotherapeutic approach that compels the modulation of tumor-associated immunosuppression and stimulates TADCs to effectively prompt anti-tumor immunity [156,157]. OVs represent a novel and promising immunotherapeutic approach that naturally perform this function. The anti-viral immune response that follows OV infection occurs within the vicinity of the tumor (*i.e*., the TME) and overturns tumor-associated immune evasion mechanisms and enhances DC activation, maturation, and TAA/TSA uptake and presentation.

OVs are attenuated, mutated, or naturally benign viruses that preferentially target and lyse cancer cells while leaving normal, non-transformed cells relatively unharmed. Currently, there are numerous prominent examples of these OVs including reovirus [158], vesicular stomatitis virus (VSV) [159], vaccinia virus [160], Newcastle disease virus [161], measles virus [162], poliovirus [163], HSV [164], coxsackievirus [165], adenovirus [166], and Maraba virus [167,168]. OVs preferentially target cancer cells as a result of aberrant cellular signaling (e.g., Ras signaling [158,169]) and defective anti-viral immune responses (e.g., type I IFNs [170,171]), which result in a weakened anti-viral immune environment within the tumor. Interestingly, the therapeutic administration of OVs drives two contrasting immunities: anti-viral and anti-tumor. Anti-tumor immunity is highly desirable whereas anti-viral immunity is often unwanted as it inhibits viral replication and spread. Hence, it is of the utmost importance to understand the interactions between DCs and OVs in the context of the TME to further improve the therapeutic efficacy of OV-based anti-cancer therapy.

### 5.1. OV-Mediated DC Activation, Maturation and Antigen Presentation

As a result of being a foreign pathogen, OV exposure naturally mediates an increased expression of a type I IFN response and a subsequent pro-inflammatory immune response to contain and eradicate the virus. In addition to a cytokine-mediated response, numerous OVs are also potent inducers of class I MHC pathway-related molecules [11,172], thus immediately providing possible tumor/viral immune recognition. With respect to DCs, OVs, such as reovirus, HSV [173], vaccinia [174], and measles virus [175,176], induce the production of numerous cytokines (e.g., reovirus specifically drives the production of GM-CSF, IL-1α, IL-6, IL-12p40/70, MCP-1, M-CSF, MIG, MIP-1α, RANTES, and TNFα by human myeloid DCs [177]); and increase the expression of co-stimulatory molecules (CD80 and CD86) and class II MHC [172]. In addition to the natural capabilities of OVs to alter the maturation status of DCs, studies on engineered OVs (e.g., adenovirus, HSV, arbovirus, poxvirus) have also focused on enhancing the interaction of OVs with DCs by encoding growth factors (GM-CSF and Flt3L), chemokines (CCL2), cytokines (IL-12, RANTES, and IFN-β), and defensins (β-defensin-2) within the viral genome. For example, the administration of E1B-deleted oncolytic adenovirus expressing β-defensin-2 (Ad-BD2-E1A) has resulted in the selective recruitment and activation of pDCs, and thus improved the type I IFN response within the TME [178]. Additionally, an oncolytic adenovirus encoding MIP-1α and Flt3L has been constructed to promote DC recruitment and expansion *in vivo*, which ultimately had a strong synergistic effect on the infiltration of tumors by DCs and T cells [179]. The administration of IL-12 and GM-CSF-expressing adenovirus (Ad-ΔB7/IL12/GMCSF) in combination with DCs in B16-F10 melanoma tumor-bearing mice also showed increased DC migration to draining lymph nodes due to the upregulation of CCL21+ lymphatic vessels around tumor tissues [180]. Greater tumor growth inhibition, increased numbers of CD4+ and CD8+ T cells, and increased CD86 expression were also observed in tumors. Similar results were obtained following a combinatory treatment of IL-12 and 4-1BBL-expressing adenovirus (Ad-ΔB7/IL-12/4-1BBL) and DCs [181]. As another case in point, intratumoral injections of HSV-1 expressing GM-CSF, also known as Talimogene laherparepvec (T-VEC), has been shown to trigger the development of anti-tumor immunity in metastatic melanoma patients [182]. This is achieved through DC stimulation (attraction and maturation) via GM-CSF, resulting in enhanced priming of antigen-specific T cells. Another example is JX-594, also known as Pexa-Vec. Intravenous delivery of this GM-CSF-expressing vaccinia poxvirus with a deletion of the thymidine kinase gene resulted in increased tumor-infiltrating CD8+ T cells and reduced metastasis of hepatocellular carcinoma [183]. Most importantly, OV-driven accumulation, activation, and heightened co-stimulatory molecule expression of DCs from OV-treated tumor-bearing hosts overturn the dysfunctional nature of TADCs and have the potential to establish a robust anti-tumor specific immune response.

In addition to the natural interaction of DCs with OVs following co-culture or therapeutic administration, studies have also shown that the incorporation of OVs with common DC-based immunotherapeutic approaches bear enhanced efficacy. For example, a DC-based vaccination approach that loaded DCs with an OV, M protein mutant oncolytic VSV (ΔM51-VSV) encoding a tumor-associated antigen, enhanced the activation, maturation, and function of DCs, and subsequently controlled tumor growth by the engagement of both NK and CD8+ T cells [184]. A similar outcome was observed when DCs loaded with measles virus-infected mesothelioma cells induced spontaneous DC maturation and significant tumor-specific CD8+ T cell proliferation [175,185]. These and other studies suggest that OVs enhance or restore DC responsiveness which potentially favors the development of anti-tumor immunity during direct oncotherapy or supplementation of OVs to an existing immunotherapeutic approach.

### 5.2. DC-Based Delivery of OVs

From the perspective of viral perseverance, DCs are now being recognized as possible cell carriers to protect against and postpone OV eradication. Mechanisms such as neutralizing antibodies, nonspecific organ (spleen, lung, and liver) or vasculature accumulation, and scavenging immune cells represent major obstacles for delivering OVs to the tumor cells [186]. As opposed to intratumoral OV injections, systemic viral administration provides a higher probability of OVs reaching metastatic tumors or multi-nodular tumors. Thus, multiple steps are now being attempted to improve the systemic delivery of OVs with the use of immune cell carriers (extensively reviewed by Roy *et al.* [186] and Willmon *et al.* [187]). Of the numerous immune cell types being evaluated (e.g., MDSCs, T cells, or macrophages), DCs have been shown to be an effective cell carrier for both oncolytic reovirus [188,189] and measles virus [190], where DCs internalized the virus thereby protecting it against neutralizing antibodies. In particular, therapeutic administrations of reovirus in previously reovirus-exposed hosts have been shown to be ineffective; however, when DCs were loaded with reovirus, enhanced survival of melanoma-bearing mice and robust anti-tumor as well as anti-viral immune responses were observed [191]. Hence, utilizing immune cells such as DCs as cell carriers provides a means to enhance systemic dissemination of OVs to reach primary and metastatic tumors, especially for OVs for which the host is likely to have pre-existing anti-viral immunity due to previous exposure.

Ultimately, the increased delivery of OVs into the TME results in enhanced oncolysis and overturning of immunosuppression. As a result, DC function is improved in two important ways that facilitates the development of effective anti-tumor immunity. First, OV-induced lysis of cancer cells releases tumor antigens, as well as other “danger” signals, that are detected by DCs [174]. While decreased MHC expression on tumor cells previously made these cells poorly immunogenic in order to avoid immune detection, the presence of OVs now allows DCs to recognize, capture, and present tumor antigens for the activation of tumor-specific CD8+ T cells. Second, the inflammatory response triggered by an OV infection overturns the dysfunction of DCs caused by tumor-mediated immunosuppression [177]. In contrast to the immature, inhibitory DCs found in the TME, DCs in the presence of OVs are fully functional and capable of activating T cells with effective co-stimulation. Therefore, these changes create a proper environment for the development of tumor-specific T cell responses during OV-based anti-cancer therapy, specifically restoring the three signals provided by DCs for the activation of T cells.

However, it is also important to note that not all interactions between OVs and DCs are synergistic. For example, oncolytic treatment with VSV has been shown to have negative effects on TADC number and function [192]. While the administration of recombinant Flt3L alone increased DC number, combining Flt3L with VSV treatment abrogated this effect. VSV directly infected and killed TADCs, thus decreasing the number of TADCs. There was also reduced tumor antigen presentation *in vivo* and decreased migration of DCs to draining lymph nodes. Therefore, there are instances where OV administration can negate DC function and effectively hamper the development of anti-tumor immunity. It remains to be shown whether these effects are OV-specific, in which case further understanding of DCs in the context of different OV types is required to optimize DC-mediated induction of anti-tumor immunity and oncolytic virotherapy.

## 6. Conclusions

**Figure 1:**
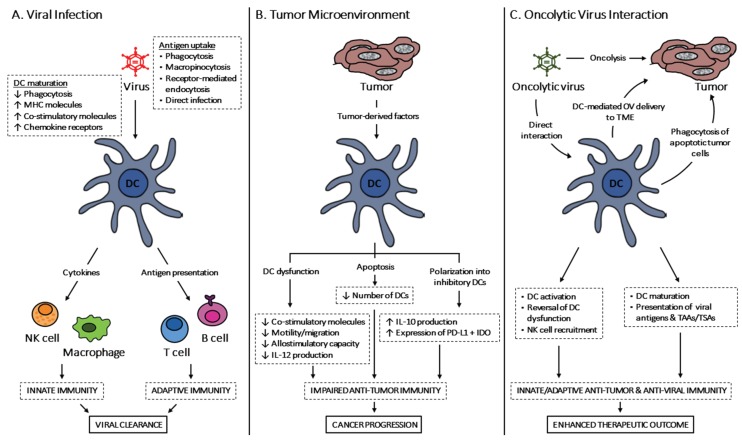
Multifunctional dendritic cells in the generation of anti-viral and anti-tumor immunity. (**A**) Upon recognition of viral infections, DCs acquire viral antigens through various mechanisms and become activated into a matured state that is more adept at antigen presentation. These DCs induce innate immune responses, in cells such as NK cells and macrophages, through the secretion of cytokines and chemokines, and also establish an anti-viral state in cells via type I IFNs. The activated DCs also prime antigen-specific T cells for the induction of adaptive immunity by presenting viral antigens in MHC molecules, along with co-stimulatory molecules and inflammatory cytokines. Overall, DCs are critical in the establishment of anti-viral immunity that eradicates the invading viral pathogen; (**B**) In the immunosuppressive TME, abnormal DC number and function hinder the generation of anti-tumor immunity. Tumor-derived factors, such as gangliosides and TGF-β, can prevent DC maturation and migration, which impede the activation of allogeneic tumor-specific T cells. DCs can also be polarized to an inhibitory state with increased PD-L1 and IDO expression, triggering T cell tolerance. Furthermore, tumor-induced apoptosis of tumor-associated, as well as circulating, DCs contribute to tumor immune evasion, thereby posing a challenge to anti-tumor immune responses; (**C**) During therapeutic OV administration, the interaction between DCs and OVs can enhance the anti-tumor effects of each constituent. While OVs can directly induce lysis of cancer cells and anti-tumor immunity, these effects can be enhanced through DC-mediated delivery of OVs to the TME, which aids OVs in avoiding anti-viral mechanisms. In return, OVs can trigger DC maturation and overturn impaired antigen presentation, thus overcoming tumor-associated immunosuppression. Moreover, OV-induced apoptosis of cancer cells releases tumor antigens, which can be captured and presented by DCs for the activation of tumor-specific T cells.

Herein, we have reviewed the role of DCs in viral infections and cancer, highlighting their capacity to generate an immune response (summarized in Figure 1). Upon detecting infectious agents or transformed cells, DCs activate immune cells to initiate anti-viral or anti-tumor immunity, respectively; both of which are associated with OV-based anti-cancer therapy. Thus, understanding the contributions of DCs to OV-driven anti-cancer responses is of the utmost importance, and the knowledge can be used to dampen the detrimental anti-viral immunity or enhance the beneficial anti-tumor immunity. By further elucidating the interaction between DCs and OVs, one can harness the DC-OV synergy to overcome the limitations of each constituent. That is, DCs can deliver OVs to the TME aiding OV-mediated oncolysis and the generation of anti-tumor immunity; while OVs can overturn impaired antigen presentation of DCs, promote DC maturation, and recruit more immune cells to the TME. Methods that can enhance DC function, such as the addition of immunostimulatory molecules or innate immune agonists, should be further explored, particularly during the administration of OVs that can negatively interfere with DC function. Ultimately, the therapeutic goal is to increase patient prognosis and survival by enhancing the efficacy of OV-based onco-immunotherapy, which may be best achieved through a combination therapy consisting of DCs and OVs.
